# Supplementary material for: How do people with disabilities in three regions of Guatemala make healthcare decisions? A qualitative study focusing on use of primary healthcare services
Source: PLOS Glob Public Health. 2023 Feb 10;3(2):e0000709. doi: 10.1371/journal.pgph.0000709 (PMC10021648; doi:10.1371/journal.pgph.0000709)
Supplement: Attachment — Submitted filename: Response to Reviewers .docx [file pgph.0000709.s002.docx]

Dear Dr. Manish Barman,

I want to thank you and your team for the feedback regarding my manuscript entitles “How do people with disabilities in Guatemala make healthcare decisions? a qualitative study focusing on use of primary healthcare services”. Below you will find my responses to each of the points raised by the editors and reviewers.

Responses are highlighted in yellow.

Please do not hesitate to reach out to me for any further clarification needed.

Sincerely,

Goli Hashemi,OTR/L, MSc. PhD Candidate

[Goli.hashemi@lshtm.ac.uk](mailto:Goli.hashemi@lshtm.ac.uk)

+1-214-908-6447

Journal Requirements:

1. Please insert an Ethics Statement at the beginning of your Methods section, under a subheading 'Ethics Statement'. It must include:

a) The name(s) of the Institutional Review Board(s) or Ethics Committee(s)

b) The approval number(s), or a statement that approval was granted by the named board(s)

An Ethics statement is now added at the beginning of the methods section with the above details (lines 144-152)

2. Please include a complete copy of PLOS’ questionnaire on inclusivity in global research in your revised manuscript. Our policy for research in this area aims to improve transparency in the reporting of research performed outside of researchers’ own country or community. The policy applies to researchers who have travelled to a different country to conduct research, research with Indigenous populations or their lands, and research on cultural artefacts. The questionnaire can also be requested at the journal’s discretion for any other submissions, even if these conditions are not met.  Please find more information on the policy and a link to download a blank copy of the questionnaire here: https://journals.plos.org/globalpublichealth/s/best-practices-in-research-reporting. Please upload a completed version of your questionnaire as Supporting Information when you resubmit your manuscript.

A completed PLOS’ questionnaire on inclusivity in global research is now added as supporting information

3. Please upload all main figures as separate Figure files in .tif or .eps format only and removed from manuscript file. For more information about how to convert and format your figure files please see our guidelines:

<https://journals.plos.org/globalpublichealth/s/figures>

All 4 figures in the manuscript have been converted to tif and uploaded as separate figures.

4. Since your data is not available for proprietary reasons, please explain via email why the data is not available. Please also include the contact information for the third party organization that should be contacted should other researchers want to request access to this data and please include the full citation of where the data can be found. We also request that you verify with us via email that any researcher will be able to obtain the data set in the same manner that the you have obtained it. If you feel you are unwilling or unable to adhere to this policy, please explain your reasons by return email and your exemption request will be escalated to the editor for approval. Your exemption request will be handled independently and will not hold up the peer review process, but will need to be resolved should your manuscript be accepted for publication. One of the Editorial team will be in touch if they require more information.

This email will be sent separately after the resubmission has been completed.

Please review your reference list to ensure that it is complete and correct. If you have cited papers that have been retracted, please include the rationale for doing so in the manuscript text, or remove these references and replace them with relevant current references. Any changes to the reference list should be mentioned in the rebuttal letter that accompanies your revised manuscript. If you need to cite a retracted article, indicate the article’s retracted status in the References list and also include a citation and full reference for the retraction notice.

References have been reviewed for ensuring they are complete and corrected. There are no citations of retracted papers in the manuscript.

Additional Editor Comments (if provided):

Dear Authors
Excellent article overall
You should try incorporate some minor changes as suggested by the reviewers.
If possible try a professional editor for easy readability and flow of words.
Looking forward to your revision.


Regards


[Note: HTML markup is below. Please do not edit.]

Reviewers' comments:

Reviewer's Responses to Questions

**Comments to the Author**

1. Does this manuscript meet PLOS Global Public Health’s [publication criteria](https://journals.plos.org/globalpublichealth/s/criteria-for-publication)? Is the manuscript technically sound, and do the data support the conclusions? The manuscript must describe methodologically and ethically rigorous research with conclusions that are appropriately drawn based on the data presented.

Reviewer #1: Yes

Reviewer #2: Yes

2. Has the statistical analysis been performed appropriately and rigorously?

Reviewer #1: Yes

Reviewer #2: Yes

3. Have the authors made all data underlying the findings in their manuscript fully available (please refer to the Data Availability Statement at the start of the manuscript PDF file)?

The [PLOS Data policy](https://journals.plos.org/globalpublichealth/s/data-availability) requires authors to make all data underlying the findings described in their manuscript fully available without restriction, with rare exception. The data should be provided as part of the manuscript or its supporting information, or deposited to a public repository. For example, in addition to summary statistics, the data points behind means, medians and variance measures should be available. If there are restrictions on publicly sharing data—e.g. participant privacy or use of data from a third party—those must be specified.

Reviewer #1: Yes

Reviewer #2: Yes

4. Is the manuscript presented in an intelligible fashion and written in standard English?

PLOS Global Public Health does not copyedit accepted manuscripts, so the language in submitted articles must be clear, correct, and unambiguous. Any typographical or grammatical errors should be corrected at revision, so please note any specific errors here.

Reviewer #1: Yes

Reviewer #2: Yes

5. Review Comments to the Author

Please use the space provided to explain your answers to the questions above. You may also include additional comments for the author, including concerns about dual publication, research ethics, or publication ethics. (Please upload your review as an attachment if it exceeds 20,000 characters)

Reviewer #1: 1) Overall, this study identified a clear research gap and generated four major themes which addresses the gap in the decision making process and its key determinants for accessing primary health services by persons living with disability (PWD) in 3 regions of Guatemala.

2) Guatemala has 22 departments across 8 regions. This qualitative survey was conducted in only 3 regions and as such the findings cannot be generalized for the entire country. The country has 10% of its 18 million population categorized as disabled with 1 in 3 households having a person living with disability. Therefore, interviewing 27 PWD cannot reflect the entirety of Guatemala. I suggest that the title and methods should reflect ‘’….in three regions of Guatemala’’ and not just Guatemala.

As per the reviewer’s recommendation, the title of the paper is now updated to indicate that it applied to “Three” regions of Guatemala. See line 3 on title page.

3) Put lines 287-292 in italics to reflect the quote and same for lines 311-312, 315-316 and 317-319

The quotes in the above lines have now been italicized. The line numbers are now different due to other changes in the paper and they occur in the following lines: 296-301; 320-322;323-325;and 325-328.

Reviewer #2: Comments:
Dear authors your manuscript is well designed and well drafted I appreciate it.
I have only two comments on your manuscript.
¬ Add citation in your discussion part by referring the previous articles to make your study more strong.

Thank you for your suggestion. The reference has now been added- line 422.

¬ Figure 1 and 2 are not visible, try to make it visible.

I am submitting the figures as separate Tif files on the platform.

In general this manuscript needs minor revision.

6. PLOS authors have the option to publish the peer review history of their article ([what does this mean?](https://journals.plos.org/globalpublichealth/s/editorial-and-peer-review-process#loc-peer-review-history)). If published, this will include your full peer review and any attached files.


**Do you want your identity to be public for this peer review?** If you choose “no”, your identity will remain anonymous but your review may still be made public.


For information about this choice, including consent withdrawal, please see our [Privacy Policy](https://www.plos.org/privacy-policy).

Reviewer #1: No

Reviewer #2: No

[NOTE: If reviewer comments were submitted as an attachment file, they will be attached to this email and accessible via the submission site. Please log into your account, locate the manuscript record, and check for the action link "View Attachments". If this link does not appear, there are no attachment files.]

While revising your submission, please upload your figure files to the Preflight Analysis and Conversion Engine (PACE) digital diagnostic tool, https://pacev2.apexcovantage.com/. PACE helps ensure that figures meet PLOS requirements. To use PACE, you must first register as a user. Registration is free. Then, login and navigate to the UPLOAD tab, where you will find detailed instructions on how to use the tool. If you encounter any issues or have any questions when using PACE, please email PLOS at figures@plos.org. Please note that Supporting Information files do not need this step.
