# Supplementary material for: How do people with disabilities in three regions of Guatemala make healthcare decisions? A qualitative study focusing on use of primary healthcare services
Source: PLOS Glob Public Health. 2023 Feb 10;3(2):e0000709. doi: 10.1371/journal.pgph.0000709 (PMC10021648; doi:10.1371/journal.pgph.0000709)
Supplement: Attachment — Submitted filename: Response to editors 21-10-22.docx [file pgph.0000709.s003.docx]

Dear Dr. Manish Barman,

# I want to thank you and your team for the ongoing feedback regarding my manuscript entitled “How do people with disabilities in three regions of Guatemala make healthcare decisions? a qualitative study focusing on use of primary healthcare services”. Below you will find my responses to the recent request for further minor revisions related to the figures 1-3 as raised by the editor.

Responses are highlighted in yellow.

Please do not hesitate to reach out to me for any further clarification needed.

Sincerely,

Goli Hashemi, OTR/L, MSc. PhD Candidate

[Goli.hashemi@lshtm.ac.uk](mailto:Goli.hashemi@lshtm.ac.uk)

+1-214-908-6447

Additional Editor Comments (if provided):

Dear Authors
The revision is satisfactory except-
The Figures now added as separate tiff files are still quite unclear and hazy.
It seems the Figures are not original by the authors and are probably sourced from somewhere.
This might have copyright issues both for you and the journal.
Either these figures should be properly referenced after taking permission from the original sources or an alternative way can be if Authors can draw /create there own figures with some modifications.

I have adapted and modified all the figures, while also referencing the original, and resubmitted them with new titles to demonstrate the adaption in the manuscript. I hope they are now more clear and there are no concerns related to copyright issues for figures 1-3.

Looking forward to your re submission ASAP
Thanks

Editor
