# Supplementary material for: How do people with disabilities in three regions of Guatemala make healthcare decisions? A qualitative study focusing on use of primary healthcare services
Source: PLOS Glob Public Health. 2023 Feb 10;3(2):e0000709. doi: 10.1371/journal.pgph.0000709 (PMC10021648; doi:10.1371/journal.pgph.0000709)
Supplement: Attachment — Submitted filename: response to reviewers 1-11-23.docx [file pgph.0000709.s004.docx]

Dear Dr. Ferdinand Mukumbang,

I want to thank you for your additional feedback and request for clarification regarding lines 222-224 of my manuscript entitled “How do people with disabilities in Guatemala make healthcare decisions? a qualitative study focusing on use of primary healthcare services”.

I have now paraphrased the statement to hopefully further clarify what was done and ease your concern regarding the handling of the qualitative data and what I meant to describe as part of the data analysis process. My response is highlighted in yellow.

Please do not hesitate to reach out to me for any further clarification needed.

Sincerely,

Goli Hashemi,OTR/L, MSc. PhD Candidate

[Goli.hashemi@lshtm.ac.uk](mailto:Goli.hashemi@lshtm.ac.uk)

+1-214-908-6447

In the data analysis section, Lines 222-224, the authors wrote: "The results were then shared with MW, who reviewed 20% of the interviews for interrater coding and discussion as necessary to ensure agreement and consensus."

This sentence is highly charged and unclear to me vis-a-vis the trustworthiness that is expected when handling qualitative data. The phrase "reviewed 20% of the interviews for interrater coding" was most confusing and I struggled to understand what the authors did practically and what the implications are regarding the trustworthiness of the study. I also wondered if having the knowledge of 20% of the interview content can lead to a meaningful discussion "to ensure agreement and consensus.

What the above statement meant to indicate was that MW independently reviewed and coded 6 of the 27 transcripts to ensure similarities in coding between GH and MW. This process also helped with further discussion about the potential themes between the authors, as it familiarized MW with at least some of content of the interviews. The section is now paraphrased to the following: *In addition to the coding performed by GH, six randomly selected transcripts were shared with MW, who also coded them independently. This process allowed for further validation of the inductive coding conducted by GH of those transcripts and MW to become somewhat familiar with the content of the interviews for additional opportunity for GH and MW to discuss the content and the categorization into themes.*

Please submit your revised manuscript by 15/01/2023. If you will need more time than this to complete your revisions, please reply to this message or contact the journal office at globalpubhealth@plos.org. When you're ready to submit your revision, log on to https://www.editorialmanager.com/pgph/ and select the 'Submissions Needing Revision' folder to locate your manuscript file.

Please include the following items when submitting your revised manuscript:

- A rebuttal letter that responds to each point raised by the editor and reviewer(s). You should upload this letter as a separate file labeled 'Response to Reviewers'.
- A marked-up copy of your manuscript that highlights changes made to the original version. You should upload this as a separate file labeled 'Revised Manuscript with Track Changes'.
- An unmarked version of your revised paper without tracked changes. You should upload this as a separate file labeled 'Manuscript'.

Guidelines for resubmitting your figure files are available below the reviewer comments at the end of this letter.

We look forward to receiving your revised manuscript.

Kind regards,

Ferdinand Mukumbang, PhD
Academic Editor
PLOS Global Public Health
